# Pirates of Clinical Trials

**DOI:** 10.7759/cureus.47819

**Published:** 2023-10-27

**Authors:** Joseph Pergolizzi, Claudio Pergolizzi, Jo Ann K LeQuang

**Affiliations:** 1 Clinical Trials, NEMA Research Inc., Naples, USA; 2 Medicine, NEMA Research Inc., Naples, USA; 3 Scientific Communications, NEMA Research Inc., Naples, USA

**Keywords:** patient recruitment, clinical study subjects, sponsors, clinical trials, contract research organization

## Abstract

With the burgeoning numbers of clinical trials, the competition among sponsors for research subjects has grown intensely. Many clinical trials fail to meet their recruitment goals. Contract research organizations (CROs) that help conduct all or portions of a clinical study have transitioned from highly specialized niches, such as biostatistical analysis or regulatory compliance, to more overall functions to keep a trial moving forward. CROs establish agreements with sponsors, including how much a site will be paid per study subject. CROs are locked into that pricing, but over the course of a study's recruitment period, sponsors with deeper pockets may step in and offer more compensation per subject. The result is a competitive market place that favors big sponsors and puts smaller CROs and start-ups at a disadvantage.

## Editorial

**Figure 1:**
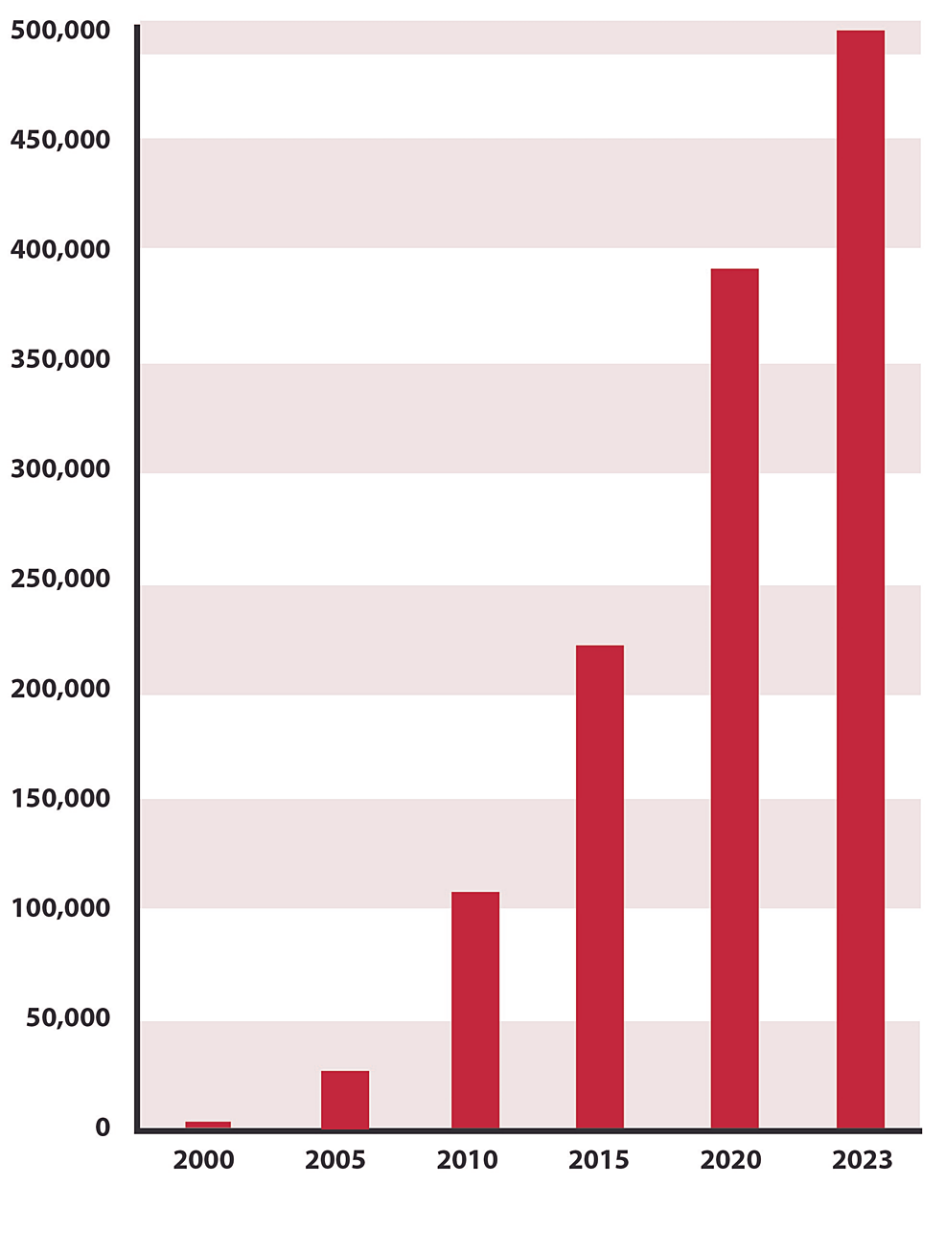
The number of registered clinical trials is rapidly increasing. Data shown for 2023 incorporate only data through May 2023.

Clinical trials are the cornerstone of modern medical research and are crucial to market clearance for new drugs, therapeutics, devices, and treatments. The number of registered clinical trials being conducted around the world has increased from just over 2,100 in the year 2000 to a staggering 454,000 by mid-2023, an increase of over 200-fold in just over two decades [1]. This has engendered such a ferocious, cut-throat competition among sites and sponsors for subjects that it jeopardizes the serious scientific work of rigorous clinical investigation (Figure 1).

A study evaluating 114 trials conducted between 1994 and 2002 found that only 31% of trials recruited all of the subjects they sought and 45% failed to recruit even within 80% of this target number [2]. There has been curiously little investigation as to the optimal recruitment strategies, allowing a business-oriented strategy to emerge, namely, the sponsor who pays most wins.

Contract research organizations (CROs) emerged when pharmaceutical sponsors sought to outsource specific and highly limited aspects of a clinical trial to a third party, allowing CROs to become niched and hyper-specialized; for example, there were CROs that specialized in biostatistics or regulatory compliance. The breakneck expansion of clinical trials has expanded the role of the mainstream CROs from a highly specialized contract agency to a more full-service role. CROs today may be called upon to select investigators, choose sites, train personnel, produce recruitment and educational content, surveil the sites for compliance, compile reports, and audit sites as needed, as well as manage data, analyze biostatistics, and draft reports for regulatory bodies. Standing between the sponsor and the regulatory body, CROs must be strictly vigilant to observe the sponsor’s protocol, federal or national regulations, its own operating procedures, and industry guidelines. CROs are thus subject to the regulatory requirements that the sponsor and agencies impose, but they must also try to adhere to timelines set by a sponsor [3].

Recruitment has emerged as such a powerful issue in clinical trials that many studies are being offshored to overcome the American reticence to participation in a clinical trial, although international trials impose an additional layer of complexity to studies, including translation, international regulations, and cultural considerations. The bioethics of international trials suggests that the way certain trials are presented to local populations may encourage what might be considered “nonconsensual participation” in that individuals may agree to participate to obtain healthcare that would otherwise not be available to them [4].

Clinical trial participants are viewed as a precious commodity in studies based in the United States. They are scarce because in some cases, sites do not proactively recruit or even consider that a patient might be interested in a trial. Subjects can be fearful of being a “guinea pig,” losing control of their medical care, or being assigned the placebo group. Strict protocols, unusual requirements (e.g., keeping a diary or making numerous clinic visits), and fear that they will not get personalized care are all serious roadblocks. All sponsors, CROs, and sites face these obstacles in getting volunteers to sign an informed consent. The impact of reticent individuals is profound on the pharmaceutical and medical device industries because many studies simply fail to launch.

The degree to which Americans resist clinical trials is hard to imagine for those outside the industry. Only 8% of cancer patients in America agree to participate in clinical trials, although many such trials offer opportunities to get new breakthrough therapies [5]. Minority populations are particularly reticent to sign up and may not always be actively recruited.

The recent rush on subjects is now playing out in financial terms. A CRO may contract with a sponsor to do a particular study. Studies can be fully or partially burdened, depending on the extent that the participant’s insurance reimburses for services. Based on these requirements, a CRO establishes a specific amount that it pays to the site for each subject. For example, a CRO and sponsor may agree that the CRO will pay $16,000 per completed subject; this fee would then cover all (fully burdened) or part (partially burdened) of the costs of treatment, medication, procedures, and so on. The site and its principal investigator know that each enrollment in the study will bring $16,000 from the CRO for one completed participant; ideally, this should provide reassurance to the site that costs are covered. However, another CRO or sponsor can enter the picture with a similar study and up the ante to $20,000 per subject participant. The site is under no obligation to continue to recruit and treat subjects based on the first agreement and can simply enroll subjects to the new study at the $20,000 price.

Meanwhile, the CRO is constrained from offering more money per subject participant because of the contract it signed with the sponsor. In this situation, sponsors may perceive that the CRO has somehow failed because its original contract has been outbid in the marketplace, but the system is set up so that CROs may bid only once (the original contract) and if a competing study offers more money later on, there is no remedy. A CRO caught in this predicament can try to renegotiate the contract with the sponsor, resulting in increased costs to the sponsor. This gives the last-to-the-site clinical team an advantage - they know the going rates already. And the game is risky, because that new study can be outbid by another study that comes along offering $21,000 per subject participant.

This current system places a substantial burden on small- and mid-sized CROs who struggle to provide cost-effective solutions to sponsors, particularly smaller sponsors, but who cannot control market conditions in the bidding wars for subjects. CROs must offer competitive prices to attract sponsor clients, but another sponsor with deep pockets can simply wait on the sidelines and then outbid the CRO. For multi-billion-dollar sponsors, paying a premium to win subjects makes sense, but it leaves smaller companies, start-ups, and entrepreneurs in the dust. A big pharma sponsor running its own study might simply opt to meet the market price per subject participant rather than derail their study. CROs do not have that luxury and must abide by their contract, and the only remedy is to increase per subject site fees to keep the sites engaged. This practical step to salvage a study in a bidding war can be viewed negatively by sponsors. In fact, some smaller sponsors may be unaware of how serious these price wars can be.

On a global basis, the problem of subject recruitment is a tough one to solve and likely would involve a broad-scale educational intervention about the nature of clinical trials in general and the value of participation to the individual and to the country. Site integrity can be stressed, such that sites fulfill commitments to their partners regardless of whether a better-paying partner arrives on the scene. Audits can be conducted to assure that sites do not jump from one study to the next to the next based on payments. It is practically impossible to force the site to fulfill their original agreement, as they can simply just decide to drop the lower-paying study. Why take a subject for $16,000 when you can get $20,000 for a similar study? All of these are challenging to accomplish in an era of burgeoning studies and ultra-competitive recruitment efforts.

Furthermore, the current system poses a potential theoretical risk to subjects if investigators opt to enroll subjects into the best-paying study rather than the study most suitable to meet the subject’s individual needs. Not only does that fail the “subject-centric” test for clinical trials, if exposed, but it would also further erode trust in investigative sites and clinical trials, which would then rightly be viewed as first-and-foremost money-making operations. A dental clinic that handles a high volume of oral surgeries may have numerous opportunities to conduct studies on pain relievers following third molar extractions. There is no reason for them to enroll in any but the best-paying studies. In such a tight market for clinical trial participants, the law of supply and demand may drive the price up beyond what is reasonable.

Other forms of warfare in the battle for registrations are promotional materials, educational content, and advertising. Major sponsors can afford to sink large sums into advertising, newsletters, social media campaigns, and other forms of outreach to encourage participation. Individuals inquiring about trial participation may be more persuaded by slick 3D visuals and glossy brochures than thorough information in the form of a black-and-white photocopied page. Nurses and other clinicians can be invited by sponsors to educational events or seminars or be given very polished courses as to how to recruit for a specific study. For many clinical trials, it is the front-line clinicians, such as nurses or even administrators, who first see patients and can screen for which individuals might qualify as study subjects. Thus, wooing these clinicians in educational or promotional events might cause them to favor one sponsor over another.

This is a call to awareness. Solutions must be found. It may be that future trends toward decentralized clinical trials have benefit. In a decentralized trial, patients would be able to participate in a study even if they did not live near an investigational site, but such studies depend on subject adherence. Virtual medicine may play a role in the form of “digital twins,” a digitally simulated patient that can be used to safely and inexpensively test various regimens and scenarios without having a human involved. Digital twins can also be used in simulations to aid in study design and even recruitment efforts by showing patients in virtual reality what the actual study might entail. However, it appears that as the number of clinical trials and subject requirements increase, the richest sponsors have a way to game the system.

Audits and better policies may be needed. However, in the race to the next blockbuster drug or breakthrough medical device, big sponsors are holding an advantage over mid- and smaller-sized CROs and start-up sponsors in terms of recruitment, and that means these wealthy sponsors have all the advantages in speed-to-market, which is a key driver in today’s healthcare economy.
